# Ribosomal protein L4 is a novel regulator of the MDM2-p53 loop

**DOI:** 10.18632/oncotarget.7479

**Published:** 2016-02-18

**Authors:** Xia He, Yuhuang Li, Mu-Shui Dai, Xiao-Xin Sun

**Affiliations:** ^1^ Department of Molecular and Medical Genetics, School of Medicine and the OHSU Knight Cancer Institute, Oregon Health & Science University, Portland, OR, USA; ^2^ Department of Radiation Oncology, Jiangsu Cancer Hospital, Nanjing, China

**Keywords:** RPL4, MDM2, p53, ribosome proteins, ubiquitination

## Abstract

A number of ribosomal proteins (RPs) have been shown to play a critical role in coordinating ribosome biogenesis with cell growth and proliferation by suppressing MDM2 to induce p53 activation. While how the MDM2-p53 pathway is regulated by multiple RPs is unclear, it remains to be interesting to identify additional RPs that can regulate this pathway. Here we report that ribosomal protein L4 (RPL4) directly interacts with MDM2 at the central acidic domain and suppresses MDM2-mediated p53 ubiquitination and degradation, leading to p53 stabilization and activation. Interestingly, overexpression of RPL4 promotes the binding of MDM2 to RPL5 and RPL11 and forms a complex with RPL5, RPL11 and MDM2 in cells. Conversely, knockdown of RPL4 also induces p53 levels and p53-dependent cell cycle arrest. This p53-dependent effect requires both RPL5 and RPL11, suggesting that depletion of RPL4 triggers ribosomal stress. Together, our results reveal that balanced levels of RPL4 are critical for normal cell growth and proliferation via regulating the MDM2-p53 loop.

## INTRODUCTION

Ribosome biogenesis, a cellular process producing ribosomes, is essential for cell growth and cell proliferation. However, deregulated ribosome biogenesis activity is associated with impaired control of cell growth and proliferation and contributes to human diseases. For example, haploinsufficiency of a number of ribosomal proteins (RPs) causes Diamond-Blackfan anemia, a congenital bone marrow failure syndrome characterized by chronic regenerative anemia, various degree of congenital abnormalities, and potential cancer predisposition [1, 2]. Mutations of the dyskeratosis congenita (DKC) gene cause dyskeratosis congenita, a disease characterized by premature aging, including bone marrow failure and hyperkeratosis of the skin, and an increased susceptibility to cancers [1, 2]. On the other hand, elevated activation of ribosome biogenesis and protein translation also contribute to tumorigenesis [3]. Therefore, ribosome biogenesis should be tightly regulated to coordinate with normal cell proliferation.

Among many ribosome biogenesis regulators, the tumor suppressor protein p53 has emerged as one of the central regulatory hubs. p53 is normally maintained at low levels, mainly by the oncoprotein MDM2, an ubiquitin (Ub) ligase (E3) [4, 5]. MDM2 binds to the N-terminal transactivation domain of p53 through its N-terminal p53-binding domain. Its C-terminal Ring-finger domain mediates p53 ubiquitination and proteasomal degradation [6, 7]. In response to various stress, p53 is transiently stabilized and activated to induce the expression of a number of target genes, whose protein products mediate diverse biological function, including cell cycle arrest, apoptosis and senescence [8, 9]. p53 has been shown to negatively regulate ribosome biogenesis via inhibiting the transcription of rRNAs, tRNAs and RP genes [10–12]. Meanwhile, p53 is a crucial sensor for abnormal ribosome biogenesis and ribosomal stress [13–15]. Initially, several RPs from the large subunit, including L5, L11, and L23, were identified to bind to MDM2 and suppress MDM2 activity towards p53, leading to p53 stabilization and activation [16–21]. Particularly, L5 and L11 are shown to be the key for p53 activation following ribosome stress, a cellular stress induced by perturbation of ribosome biogenesis [22–24]. Mice with a knockin of the cancer-associated MDM2 mutant, C305F (MDM2^C305F^), which fails to bind to L5 and L11, displayed a specific defect in p53 signaling in response to ribosomal stress, but not DNA damage, further validating the ribosomal stress-induced RPs-MDM2-p53 signaling pathway *in vivo* [25]. Recently, it has been shown that 5S rRNA and thus the L5-L11-5S rRNA RNP are essential for p53 activation in response to ribosomal stress [26, 27]. Likewise, increasing numbers of RPs, including L26 [28], L6 [29], S7 [30, 31], S14 [32], S25 [33], S27 [34], S27a [35], S26 [36], L37, S15 and S20 [37] were also shown to have similar function to inhibit MDM2. Yet, not all RPs have this function to bind to and inhibit MDM2 [38]. However, knockdown of some of the MDM2-interacting ribosomal proteins, such as L23 and S14, also causes ribosomal stress and induces p53 [18, 19, 32, 37], suggesting that these RPs play a redundant role in regulating the MDM2-p53 pathway. While how multiple RPs regulate the MDM2-p53 pathway remains unclear [15], it is still interesting to examine whether additional RPs can regulate the MDM2-p53 pathway.

Here, we report that RPL4 is a novel regulator of the MDM2-p53 loop. We found that RPL4 directly binds to MDM2 in cells and *in vitro* and inhibits MDM2-mediated p53 ubiquitination and degradation in cells. We further demonstrated that RPL4 promotes the RPL5-RPL11-MDM2 complex formation. On the other hand, knockdown of RPL4 triggers ribosomal stress to induce p53 and p53-dependent cell cycle arrest, which requires RPL5 and RPL11. Thus, balanced levels of RPL4 are crucial for maintaining normal levels of p53 in cells.

## RESULTS

### RPL4 interacts with MDM2 in cells and *in vitro*

**Figure 1:**
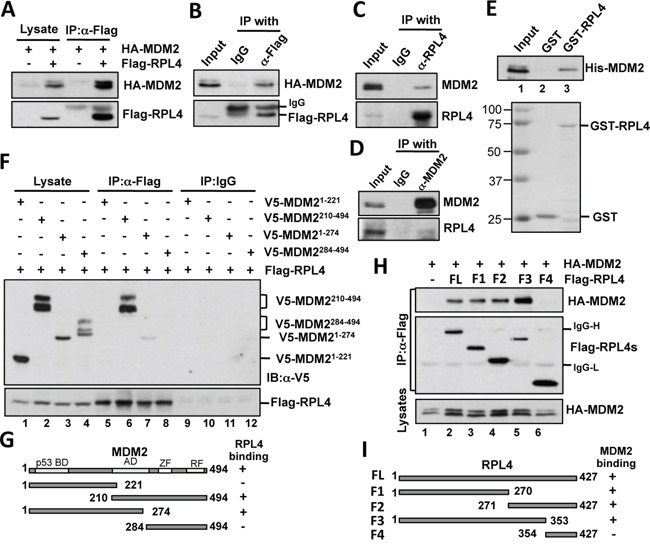
RPL4 interacts with MDM2 in cells and *in vitro* **A, B.** Exogenous RPL4 interacts with exogenous MDM2. H1299 cells transfected with HA-MDM2 alone or together with Flag-RPL4 were subjected to co-IP with anti-Flag antibody (A) or control mouse IgG (B) followed by IB assays. **C, D.** Endogenous RPL4 interacts with endogenous MDM2. U2OS cells were treated with 5 nM Actinomycin D for 8 hours. The cell lysates were immunoprecipitated with mouse monoclonal anti-L4 antibody (C), anti-MDM2 (D), or control mouse IgG, followed by IB with anti-MDM2 or anti-L4 antibodies. **E.** L4 interacts with MDM2 *in vitro*. Purified GST or GST-RPL4 immobilized on glutathione beads was incubated with recombinant His-MDM2. Bound proteins were assayed by IB using anti-MDM2 antibodies. Coomassie staining of the GST and GST-RPL4 proteins is shown in the bottom panel. **F.** RPL4 binds to the central acidic domain of MDM2. H1299 cells were transfected with Flag-RPL4 together with plasmids encoding V5-tagged MDM2 deletion mutants as indicated. Cell lysates were immunoprecipitated with an anti-Flag antibody or control mouse IgG followed by IB. **G.** Schematic diagram of MDM2 deletion mutants with the indication of the acidic domain (AD), zinc finger (ZF) domain, and ring finger (RF) domain. **H.** Mapping the MDM2 binding domain on RPL4 in cells. H1299 cells were transfected with HA-MDM2 together with Flag-RPL4 or its deletion mutants. Cell lysates were immunoprecipitated with the anti-Flag antibody followed by IB using anti-MDM2 and anti-Flag antibodies. **I.** Schematic diagram of RPL4 proteins and their binding to MDM2.

To find additional RPs that may regulate the MDM2-p53 pathway, we have tested the ability of a series of Flag-tagged RPs from the large subunit of ribosome to bind to MDM2 and found RPL4 as one of the MDM2-interacting proteins (data not shown). To further verify the interaction, we performed co-immunoprecipitation (co-IP) assays in H1299 cells transfected with MDM2 alone or together with RPL4. As shown in Figure 1A, ectopically expressed MDM2 was co-immunoprecipitated with Flag-RPL4 using anti-Flag antibody when both proteins were expressed. This interaction was specific as MDM2 was not co-immunoprecipitated by control IgG in cells co-transfected with HA-MDM2 and Flag-RPL4 (Figure 1B). Also, endogenous MDM2 was specifically co-immunoprecipitated with endogenous RPL4 by either anti-L4 antibody (Figure 1C) or by anti-MDM2 antibody (Figure 1D), but not the control IgG, in U2OS cells treated with actinomycin D. To determine whether RPL4 directly binds to MDM2, we conducted GST-fusion protein-protein association assays. As shown in Figure 1E, purified His-MDM2 was bound by purified GST-RPL4 protein, but not GST alone. These results demonstrate that RPL4 directly binds to MDM2 in cells and *in vitro*.

### RPL4 binds to the central acidic domain of MDM2

Most of the MDM2-interacting RPs bind to MDM2 at the central region containing the acidic domain and zinc finger domain [15]. To determine whether RPL4 binds to MDM2 at the similar central region, we performed transfection-co-IP experiments. H1299 cells transfected with Flag-RPL4 together with V5-MDM2 deletion mutants were assayed by co-IP using anti-Flag antibody or control mouse IgG. As shown in Figure 1F, the central acidic domain (amino acids 221 to 274) containing mutants (lanes 6 and 7) of MDM2, but not the N-terminal (lane 5) and the C-terminal (lane 8) fragments, interacted with RPL4. Thus, the central acidic domain is required for MDM2 to interact with RPL4 (Figure 1G). Similarly, we transfected cells with HA-MDM2 together with Flag-RPL4 or its deletion mutants. Co-IP using anti-Flag antibody showed that both the N-terminus (F1) and C-terminus (F2) fragments of RPL4, but not the shorter C-terminus fragment (F4), interacted with MDM2. Thus, RPL4 contains two MDM2-binding sites located in the N-terminus (1-270) and the middle region (271-353), respectively (Figure 1H and 1I).

### RPL4 suppresses MDM2-mediated ubiquitination and degradation of p53

**Figure 2:**
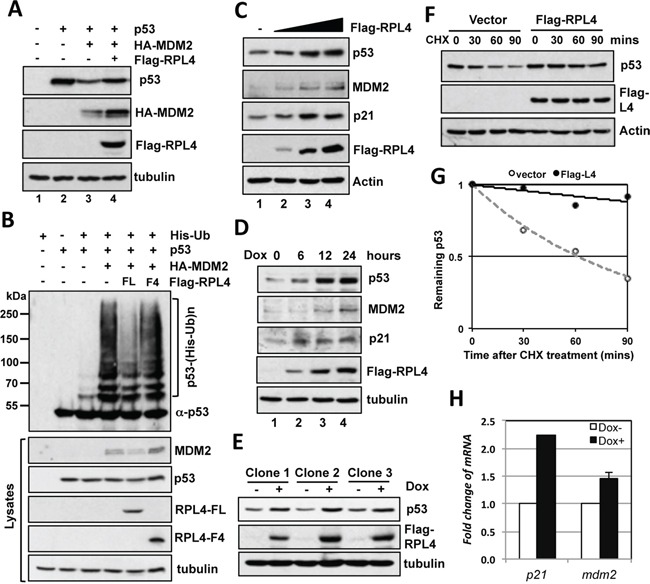
Overexpression of RPL4 inhibits MDM2-mediated p53 ubiquitination and degradation **A.** Overexpression of RPL4 inhibits MDM2-mediated p53 degradation. H1299 cells were transfected with the indicated plasmids and assayed for the expression of p53 and MDM2 by IB. **B.** RPL4 inhibits MDM2-mediated p53 ubiquitination in cells. H1299 cells transfected with combinations of the indicated plasmids were treated with MG132 (40 μM) for 6 hours before harvesting, followed by the *in vivo* ubiquitination assay. The ubiquitinated species of p53 detected by IB using the anti-p53 antibodies are indicated on right of upper panel. The expression of indicated proteins is shown in lower panels. **C.** Overexpression of RPL4 induces the levels of endogenous p53 in cells. U2OS cells transfected with the increasing amount of Flag-RPL4 plasmid were assayed by IB. **D, E.** Induced expression of RPL4 also stabilizes p53. U2OS-TO-Flag-RPL4 cells expressing tetracycline (tet)-inducible Flag-RPL4 (clone 2) were cultured in the absence or presence of doxycycline (2 μg/ml) for different time points, followed by IB detection of the indicated proteins (D). IB was also performed in three individual U2OS-TO-Flag-RPL4 clones cultured in the absence or presence of doxycycline (E). **F, G.** Overexpression of RPL4 stabilizes p53. U2OS cells transfected with control or Flag-RPL4 were treated with 50 μg/ml of CHX and harvested at different time points. The cell lysates were assayed by IB to detect the levels of the indicated proteins (F). The relative levels of p53 were normalized against the expression of actin and plotted in G. **H.** Overexpression of RPL4 induces the mRNA levels of p53 target genes. U2OS-TO-Flag-RPL4 cells were cultured in the absence or presence of doxycycline (2 μg/ml) for 24 hours. The cells were assayed for mRNA expression of the indicated genes by RT-qPCR, normalized to the expression of GAPDH.

To examine the role of RPL4 interaction with MDM2 in cells, we first performed co-transfection-immunoblot (IB) analysis in H1299 cells. As shown in Figure 2A, overexpression of RPL4 partially rescued the degradation of p53 by MDM2 (compare lane 4 to lane 3), suggesting that RPL4 inhibits MDM2-mediated degradation of p53. Consistently, overexpression of RPL4 significantly inhibited MDM2-mediated p53 ubiquitination in cells (Figure 2B). This inhibition requires MDM2-RPL4 interaction as the C-terminus fragment (F4) that does not interact with MDM2 did not inhibit MDM2-mediated p53 ubiquitination (Figure 2B). To determine whether RPL4 regulates the levels and activity of endogenous p53, we transfected RPL4 into wild-type p53-containing U2OS cells followed by IB detection of the levels of p53. As shown in Figure 2C, overexpression of RPL4 induced the p53 levels in a dose-dependent manner. Doxycycline-induced expression of RPL4 also induced the levels of endogenous p53 in U2OS cells (Figure 2D). Similar results were shown in multiple U2OS-TO-Flag-RPL4 clones (Figure 2E). Consistently, overexpression of RPL4 significantly stabilized endogenous p53 as determined by half-life assay (Figure 2F, 2G). The levels of the p53 targets p21 and MDM2 were also induced as determined by IB (Figure 2C, 2D) and RT-qPCR (Figure 2H) analysis. Together, these results suggest that overexpression of RPL4 stabilizes and activates p53 in cells.

### RPL4 interacts with the RPL5-RPL11-MDM2-p53 complex in cells

**Figure 3:**
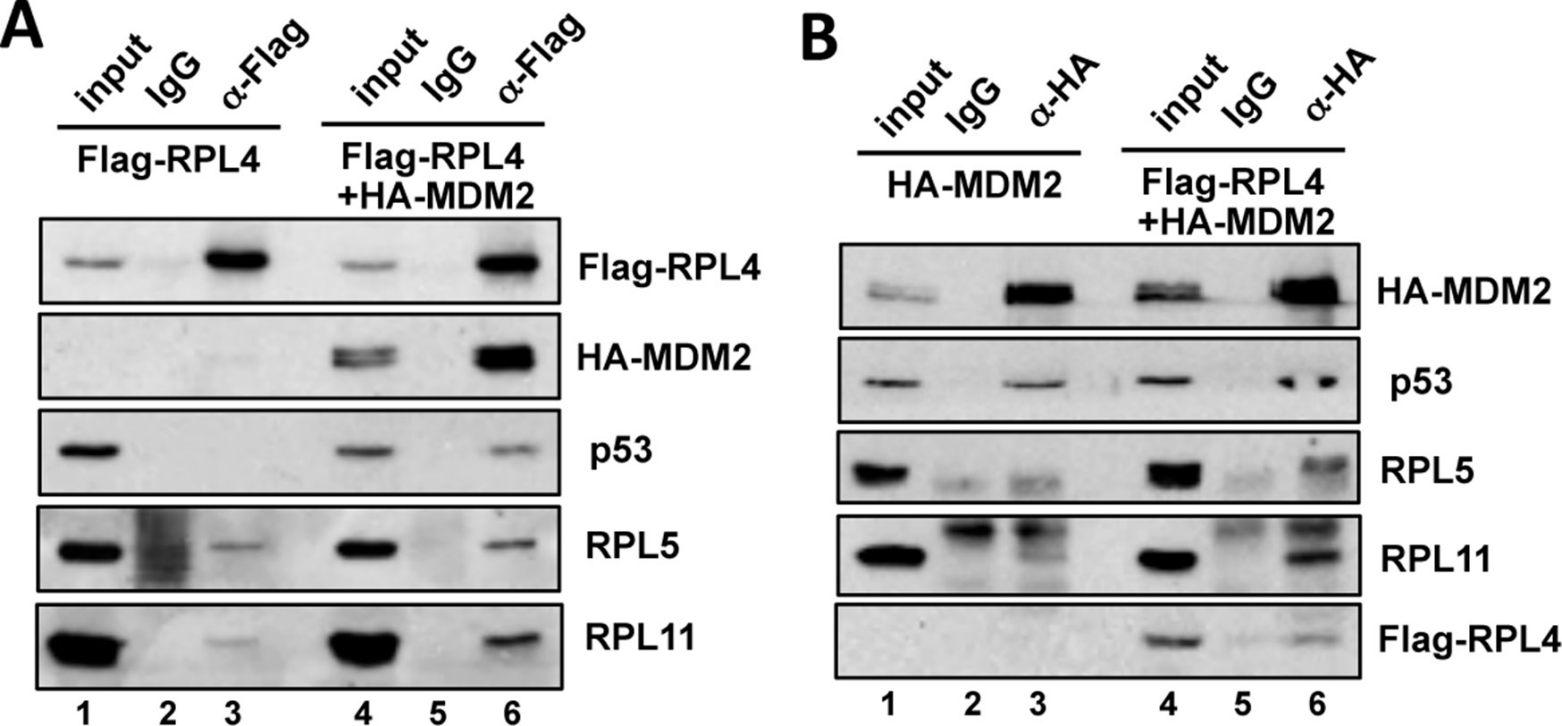
RPL4 forms a complex with MDM2, RPL5 and RPL11 and promotes the interaction of MDM2 with RPL5 and RPL11 **A.** L4 interacts with the MDM2-RPL5-RPL11 complex. U2OS cells were transfected with Flag-RPL4 alone or together with HA-MDM2. The cell lysates were immunoprecipitated with anti-Flag antibody or control mouse IgG, followed by IB. **B.** RPL4 promotes the binding of RPL5 and RPL11 to MDM2. U2OS cells transfected with HA-MDM2 alone or together with Flag-RPL4 were assayed by co-IP using anti-HA antibody or control mouse IgG, followed by IB.

A number of ribosomal proteins have been shown to interact with MDM2 and suppress its activity towards p53. Particularly RPL5 and RPL11 are required for p53 activation in cells in response to ribosomal stress [22–24]. To determine whether RPL4 could associate with this RPs-MDM2-p53 complex, U2OS cells transfected with Flag-RPL4 alone or together with HA-MDM2 were subjected to co-IP assays using anti-Flag antibody or control IgG. As shown in Figure 3A, both endogenous RPL5 and RPL11 were co-immunoprecipitated with RPL4 (lane 3). When MDM2 was co-expressed, the interaction of RPL4 with RPL5, RPL11 and p53 were significantly increased (compare lane 6 to lane 3, Figure 3A). These results suggest that RPL4 may associate with the RPL5-RPL11-MDM2-p53 complex. Interestingly, when RPL4 was co-expressed with MDM2, we found that RPL4 significantly promoted the interaction of RPL5 and RPL11 with MDM2 (compare lane 6 to lane 3; Figure 3B), suggesting that RPL4 may also suppress MDM2 and activate p53 via enhancing the RPL5 and RPL11 suppression of MDM2 besides its direct role on MDM2.

### Knockdown of RPL4 induces p53 levels and p53-dependent cell cycle arrest in cells

**Figure 4:**
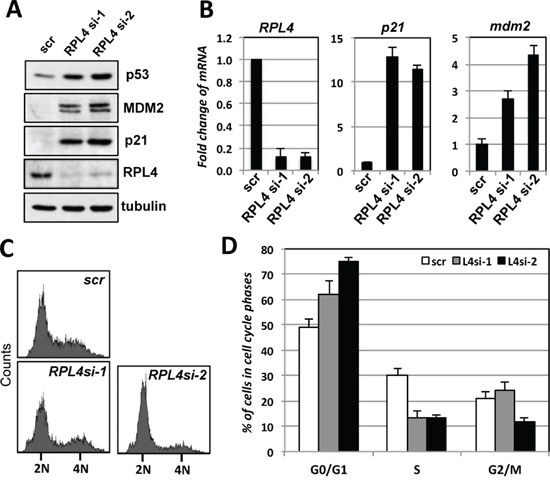
Knockdown of RPL4 induces p53 levels and G1 cell cycle arrest **A, B.** Knockdown of RPL4 induces the levels of p53 and its target genes. U2OS cells were transfected with scrambled (scr) or one of the two RPL4 siRNAs as indicated. The cell lysates were assayed for the expression of p53, MDM2 and p21 proteins using IB (A) as well as *RPL4, p21 and MDM2* mRNA using RT-qPCR, normalized to the expression of GAPDH (B). **C, D.** Knockdown of RPL4 induces G1 cell cycle arrest. U2OS cells were transfected with scrambled or one of the two RPL4 siRNAs as in (A). At 48 hours posttransfection, the cells were assayed for cell cycle profile by PI staining and flow cytometry analysis. Shown are representative histograms (C) and the summary of cell cycle phases (D). 2N and 4N indicate DNA contents of the G0/G1 (diploid) and G2/M (tetraploid) phase cells, respectively.

**Figure 5:**
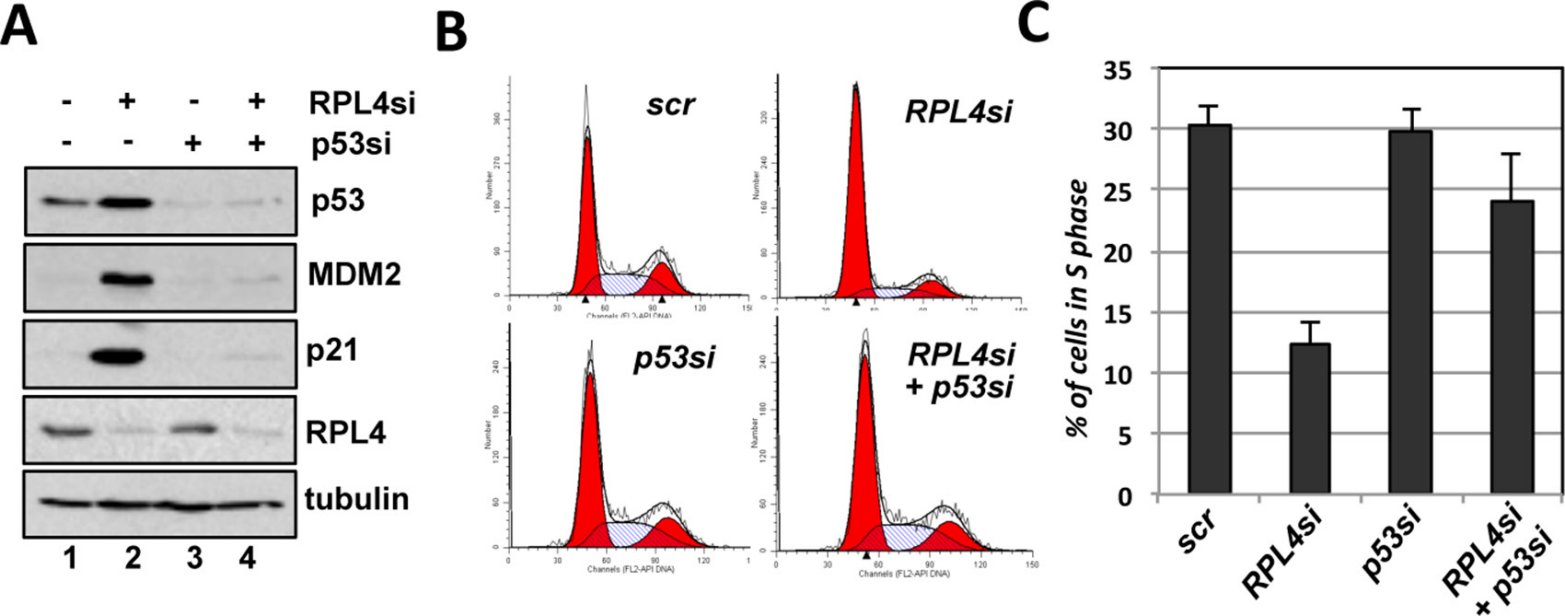
RPL4 knockdown-induced G1 cell cycle arrest is p53-dependent **A.** Knockdown of p53 abolished the induction of p21 and MDM2 by RPL4 knockdown. U2OS cells were transfected with RPL4 siRNA and p53 siRNA individually or together, followed by IB analysis. **B, C.** Knockdown of p53 abolished the RPL4 knockdown-induced cell cycle arrest. U2OS cells were transfected with L4 siRNA and p53 siRNA individually or together, followed by PI staining and flow cytometry analysis. Shown are representative histograms (B) and the summary of the percentage of S phase cells (C).

To understand the role of endogenous RPL4 in the p53 pathway, we performed siRNA-mediated knockdown of RPL4 in U2OS cells. As shown in Figure 4A, knockdown of RPL4 by two different siRNAs all drastically induced the levels of p53. The induced p53 is active as the mRNA and protein levels of the p53 target genes MDM2 and p21 were significantly induced (Figure 4A and 4B). Consistently, knockdown of RPL4 significantly increased the G1 phase cells and reduced S phase cells (Figure 4C and 4D), indicating that knockdown of RPL4 activates p53 and induces cell cycle arrest. To determine whether the G1 cell cycle arrest is p53-dependent, we performed RPL4 and p53 co-knockdown experiments. As shown in Figure 5A, knockdown of p53 completely abolished the induction of p21 and MDM2 induced by knockdown of RPL4 (compare lane 4 to lane 2). Consistently, knockdown of p53 also abolished G1 cell cycle arrest induced by RPL4 knockdown (Figure 5B and 5C). Thus, knockdown of RPL4 induces p53-dependent cell cycle arrest in cells, suggesting that knockdown of RPL4 may disrupt ribosome biogenesis and induces ribosomal stress.

### Knockdown of RPL4 results in RPL5- and RPL11-dependent p53 activation

**Figure 6:**
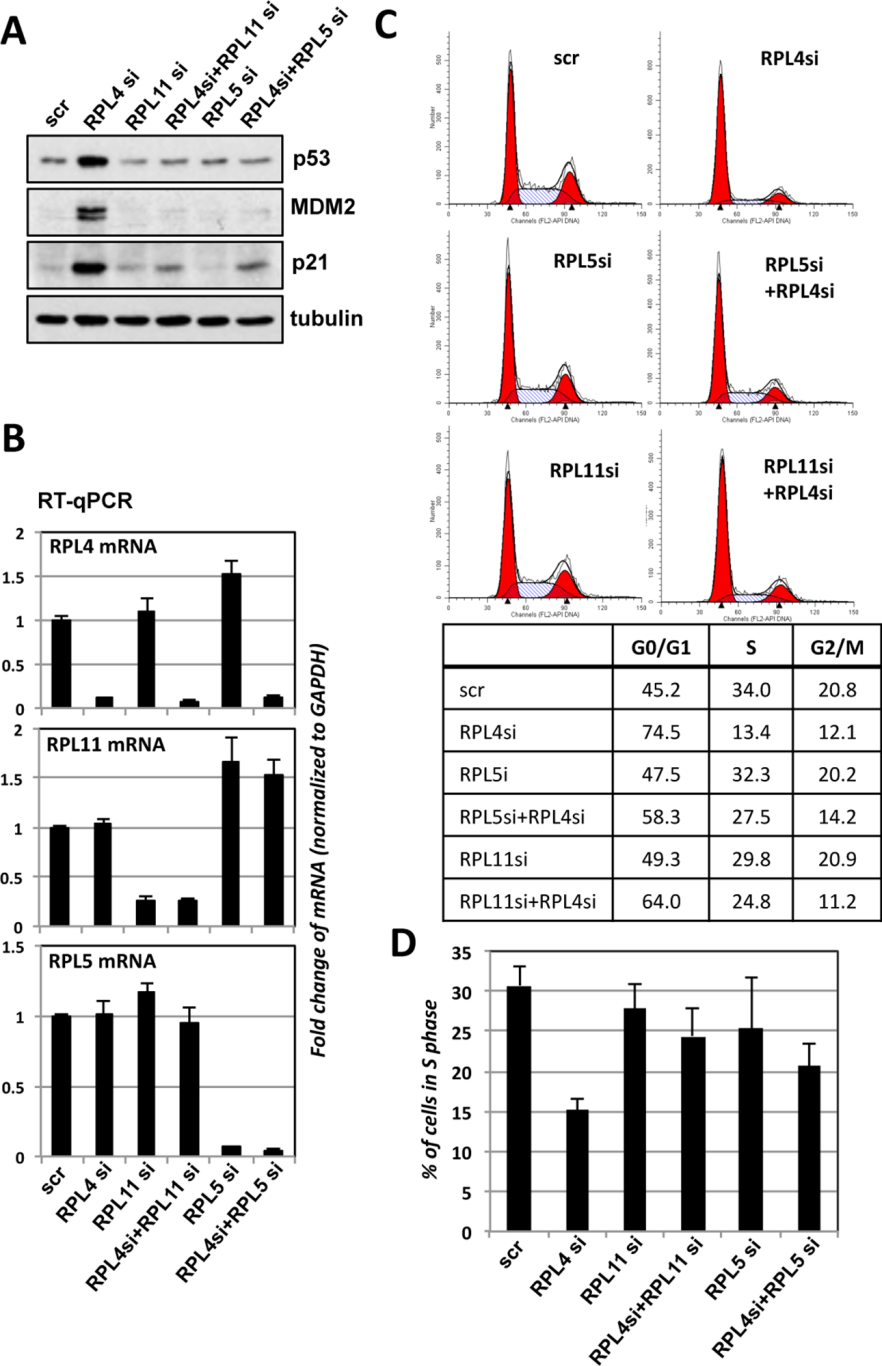
p53 activation induced by knockdown of RPL4 requires RPL5 and RPL11 **A, B.** Knockdown of RPL5 or RPL11 abolished the induction of p53 by knockdown of RPL4. U2OS cells transfected with indicated siRNAs were assayed by IB for the expression of p53, MDM2, and p21 (A). The knockdown of individual ribosomal proteins was shown in (B). **C, D.** Knockdown of RPL5 or RPL11 abolished the RPL4 knockdown-induced cell cycle arrest. U2OS cells were transfected with scrambled, RPL4 siRNA, RPL5 siRNA, or RPL11 siRNA as indicated. Cell cycle profiles were determined by PI staining and flow cytometry analysis. Shown are representative histograms (C) and the summary of the percentage of S phase cells (D).

Perturbation of ribosome biogenesis results in ribosomal stress-p53 activation signaling that is largely RPL5- and RPL11- dependent [22–24]. Thus, we asked whether the p53 induction upon RPL4 knockdown is also due to the induction of ribosomal stress and dependent on RPL5 and RPL11. As shown in Figure 6A, knockdown of either RPL5 or RPL11 abolished the p53 induction by RPL4 knockdown, and consistently, the induction of p21 and MDM2 was also abolished by knockdown of either RPL5 or RPL11. Knockdown of these individual RPs was shown in Figure 6B. Consistently, Knockdown of RPL5 or RPL11 significantly abolished the G1 cell cycle arrest induced by RPL4 knockdown (Figure 6C and 6D). Therefore, these results indicate that knockdown of RPL4 triggers ribosomal stress that activates RPL5 and RPL11-dependent p53 activation.

## DISCUSSION

**Figure 7:**
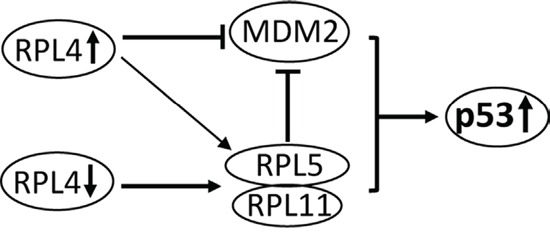
Schematic model for the role of RPL4 in regulating the MDM2-p53 signaling Bars indicate inhibition and arrows indicate activation. RPL4 directly binds to and suppresses MDM2 and also promotes the binding of RPL5 and RPL11 to MDM2, leading to p53 stabilization, whereas knockdown of RPL4 triggers ribosomal stress, leading to RPL5 and RPL11-dependent p53 activation.

In this study, we found that RPL4 is a novel regulator of the MDM2-p53 pathway. RPL4 directly binds to MDM2 both in cells and *in vitro* and significantly suppresses MDM2-mediated p53 ubiquitination and degradation, leading to p53 activation, whereas knockdown of RPL4 also significantly induces p53 activation (Figure 7). Thus, balanced levels of RPL4 are critical for maintaining normal levels of p53 and cell homeostasis.

Like many MDM2-binding RPs such as RPL23, RPL26, and RPS27 [18, 19, 39, 40], RPL4 also binds to the central acidic domain of MDM2, a region that is critical for MDM2-mediated p53 ubiquitination and degradation in cells [41–43]. By contrast, RPL5, RPL11, and RPS7 bind to the adjacent zinc finger domain of MDM2 and play an indispensable role in p53 activation in response to ribosomal stress [16, 25, 30, 44]. Mutation of Cys 305 at the zinc finger domain of MDM2 abolishes its binding to RPL5 and RPL11 in cells [44] and specifically abrogates p53 signaling in response to ribosomal stress *in vivo* [25], emphasizing the importance of the central zinc finger domain in RP regulation of the MDM2-p53 loop. Since RPL4 binds to the acidic domain of MDM2 and plays a redundant role in p53 activation, our data suggest that the acidic domain of MDM2 might also be critical for RP-regulation of the MDM2-p53 loop in response to ribosomal stress. The binding of non-redundant MDM2-binding RPs to different central regions of MDM2 suggests that these RPs may form a multi-RP-MDM2 complex and collaboratively suppress MDM2 E3 activity towards p53. Indeed, we found that RPL4 promotes the MDM2-RPL5-RPL11 complex formation and thus promotes the role of RPL5-RPL11 to further suppress MDM2 in cells. Thus, RPL4 suppresses MDM2 via both directly interaction with MDM2 and promoting the inhibition of MDM2 by RPL5 and RPL11 (Figure 7). This finding may also explain why multiple RPs are required for optimal suppression of MDM2 and suggest that the RPL5- RPL11-MDM2 might be the core RPs-MDM2 complex while other RPs like RPL4 could facilitate the optimal inhibition of MDM2 by the core RPs-MDM2 complex. Knockdown of RPL5 or RPL11 may interfere with the formation of a multi-protein complex and attenuate the p53 activation following ribosomal stress. Alternatively, overexpression of individual RPs could cause imbalance of the ribosomal component and ultimately trigger ribosomal stress as well. Also, it is possible that different RPs may respond to different stress to promote MDM2-RPL5-RPL11 complex formation.

On the other hand, we found that knockdown of RPL4 drastically induced the levels and activity of p53, suggesting that RPL4 is essential for ribosome biogenesis and its depletion may cause ribosomal stress. This notion is further supported by the observation that p53 induction and activation induced by RPL4 knockdown requires both RPL5 and RPL11, as knockdown of either RPL5 or RPL11 completely abolished the p53 induction and impaired the cell cycle arrest induced by RPL4 knockdown (Figure 6). Our results suggest that RPL4 plays an important role in the p53 signaling, yet it may not be essential for p53 activation in response to ribosomal stress. Nevertheless, balanced levels of RPL4 are required for maintaining normal physiological levels of p53, ensuring tight coordination between ribosome biogenesis and cell growth and proliferation. It is interesting in future studies to examine whether RPL4 is deregulated in human cancers and whether RPL4 regulates the MDM2-p53 pathway in vivo using animal models.

## MATERIALS AND METHODS

### Cell culture, plasmids and antibodies

Human p53-null lung non-small cell carcinoma H1299 and p53-proficient osteosarcoma U2OS cells were cultured in Dulbecco's modified Eagle's medium (DMEM) supplemented with 10% fetal bovine serum (FBS), 50 U/ml penicillin and 0.1 mg/ml streptomycin at 37°C in a 5% CO2 humidified atmosphere as described [45, 46]. Flag-tagged RPL4 expression plasmid (Flag-RPL4) was constructed by inserting full-length RPL4 cDNA into pcDNA3-2Flag vector at Bam HI and Xba I sites. The cDNA was amplified by PCR from Hela cell mRNA using primers: 5′-CGCGGATCCATGGCGTGTGCTCGCCACTG-3′ and 5′-CCGTCTAGA TTATGCAGCAGGCTTCTTCTC-3′. All the Flag-RPL4 deletion mutants were also generated using PCR and were cloned into the pcDNA3-2Flag vector. GST-RPL4 bacterial expression vector was constructed by inserting the full-length RPL4 into the pEGX.4T.1 vector (GE Healthcare). The HA-MDM2, His-MDM2, V5-tagged MDM2 (V5-MDM2) and its deletion mutant plasmids have been previously described [35, 46]. Anti-Flag (M2, Sigma), anti-p21 (Ab-11, NeoMarkers), anti-p53 (DO-1, Santa Cruz), anti-MDM2 (SMP14, Santa Cruz), anti-L4 (Santa Cruz), and anti-V5 (Invitrogen) were purchased.

### Cotransfection, immunoblot (IB) and co-immunoprecipitation (Co-IP) analyses

Cells were transfected with plasmids using TransIT^®^-LT1 reagents following the manufacturer's protocol (Mirus Bio Corporation). Cells were harvested 36-48 hours posttransfection and lysed in NP40 lysis buffer consisted of 50 mM Tris-HCl (pH 8.0), 0.5% Nonidet P-40, 1 mM EDTA, 150 mM NaCl, and 1 mM phenylmethylsulfonyl fluoride (PMSF), 1 mM dithiothreitol, 1 μg/ml pepstatin A, and 1 mM leupeptin. Equal amounts of clear cell lysate were used for IB and co-IP analyses as described previously [35, 46].

### Glutathione S-transferase (GST)-fusion protein-protein association assays

His-tagged MDM2 expressed in *E. coli* was purified through a Ni^2+^-NTA (Qiagen) column. GST-fusion protein-protein association assays were conducted as described [35, 46]. Briefly, purified His-MDM2 proteins (200 ng) were incubated with the glutathione-Sepharose 4B beads (Sigma) containing 200 ng of GST-RPL4 or GST alone. Bound proteins were assayed by IB using anti-MDM2 antibodies.

### Generation of tet-inducible RPL4 expression cell lines

To generate tet-inducible expression of RPL4, RPL4 cDNA was subcloned into pcDNA4-TO (Invitrogen) vector to generate pcDNA4-TO-Flag-RPL4 plasmid. T-Rex-U2OS cells (Invitrogen) were transfected with pcDNA4-TO-Flag-RPL4, followed by selection in culture medium containing 50 μg/ml of hygromycin and 100 μg/ml of Zeocin for 2 weeks. Single colonies were isolated, expanded, and screened by IB analysis for doxycycline (Dox)-induced expression using anti-Flag antibodies.

### RNA interference (RNAi)

RNAi-mediated gene knockdown was performed essentially as previously described (18, 47). The target sequences for RPL4 were 5′-ggccgaatgtttgcaccaa-3′ (L4 si-1) and 5′-gaagaccaaggaagctgtt-3′(L4 si-2). The control scramble sequence was described (18). All the 21-nucleotide siRNA duplexes with a 3′ dTdT overhang were synthesized (GE Dharmacon). These siRNA duplexes (100 nM) were introduced into cells using SilentFect (Bio-Rad), following the manufacturer's protocol.

### Cell cycle analysis

Cells transfected with scrambled or RPL4 siRNAs were harvested and stained in 500 μl of propidium iodide (PI, Sigma) stain buffer (50 μg/ml PI, 200μg/ml RNase A, 0.1% Triton X-100 in phosphate buffered saline) at 37°C for 30 min. The cells were then analyzed for DNA content using a Becton Dickinson FACScan flow cytometer. Data were collected using CellQuest and analyzed with the ModFit software program.

### *In vivo* ubiquitination assay

*In vivo* ubiquitination assay was conducted as previously described using Ni^2+^-NTA purification method (35, 46). Briefly, cells transfected with indicated plasmids were treated with 40 μM MG132 for 6 h before harvesting. The cells were harvested at 48 h after transfection, and 20% of the cells were used for direct IB and the rest of cells were used for ubiquitination assays under denaturing conditions using Ni^2**+**^-NTA pulldown. The bead-bound proteins were analyzed using IB.

### Reverse transcription (RT) and quantitative PCR analyses

Total RNA was isolated from cells using Qiagen RNeasy Mini Kits (Qiagen, Valencia, CA). After reverse transcriptions, quantitative (q) PCR was performed on an ABI StepOne™ real-time PCR system (Applied Biosystems) using SYBR Green Mix (Bio-Rad) as described previously [46, 48]. All reactions were carried out in triplicate. Relative gene expression was calculated using the ΔCτ method following the manufacturer's instructions. The primers used were 5′-GGGACGTTTCTGCATTTGGA-3′ and 5′-ACGCCAAGTGCCGTACAATT-3′ (RPL4); 5′-TGGAACCGTCCCAAAATGTC-3′ and 5′-GAGGAAGCTTGCCTTCTTTTGAG-3′ (RPL5) and 5′-GGGATCCAGGAACACATCGA-3′ and 5′-AGAAGTCCAGGCCGTAGATACCA-3′ (RPL11). The primers for *p21*, *mdm2*, and *GAPDH* were described [23].
